# Bi-Interfacial Electron Modulation in Co_9_S_8_/FeCoS_2_ Heterostructures Anchored on Bamboo-Derived Carbon Quasi-Aerogel for High-Performance Hydrogen Evolution

**DOI:** 10.3390/gels11060390

**Published:** 2025-05-25

**Authors:** Wenjing He, Jianliang Cao, Xinliang Zhou, Ning Zhang, Yuzhu Qi, Jin Li, Naiteng Wu, Xianming Liu

**Affiliations:** 1 School of Chemistry and Chemical Engineering, Henan Polytechnic University, Jiaozuo 454000, China; hewenjing628@126.com; 2 Henan Key Laboratory of Function-Oriented Porous Materials, College of Chemistry and Chemical Engineering, Luoyang Normal University, Luoyang 471934, China; zxlly0904@163.com (X.Z.); zn3692580147@163.com (N.Z.); qiyz5755@163.com (Y.Q.); lijin1986@lynu.edu.cn (J.L.); myclxm@163.com (X.L.)

**Keywords:** carbon quasi-aerogel, hydrogen evolution electrocatalyst, heterostructure, transition metal sulfides

## Abstract

Hydrogen energy as a sustainable alternative to fossil fuels necessitates the development of cost-effective and efficient electrocatalysts for the hydrogen evolution reaction (HER). While transition metal sulfides have shown promise, their practical application is hindered by insufficient active sites, poor conductivity, and suboptimal hydrogen adsorption kinetics. Herein, we present a heterointerface engineering strategy to construct Co_9_S_8_/FeCoS_2_ heterojunctions anchored on bamboo fiber-derived nitrogen-doped porous carbon (Co_9_S_8_/FeCoS_2_/BFPC) through hydrothermal synthesis and subsequent carbonization. BFPC carbon quasi-aerogel support not only offers a high surface area and conductive pathways but also enables uniform dispersion of active sites through nitrogen doping, which simultaneously optimizes electron transfer and mass transport. Experimental results demonstrate exceptional HER performance in alkaline media, achieving a low overpotential of 86.6 mV at 10 mA cm^−2^, a Tafel slope of 68.87 mV dec^−1^, and remarkable stability over 73 h of continuous operation. This work highlights the dual advantages of heterointerface design and carbon substrate functionalization, providing a scalable template for developing noble metal-free electrocatalysts for energy conversion technologies.

## 1. Introduction

While technological advances are improving the quality of human life, they are also facing the serious challenge of energy shortage [1]. Fossil fuels, as the backbone of traditional energy sources, are causing global concern because of their non-renewable resource properties and the environmental costs of high carbon emissions [2,3]. In this context, the development of a renewable and clean energy system has become an international consensus, in which hydrogen energy is leading the wave of the global energy revolution by virtue of its unique advantages of unlimited reserves, high energy density, and zero carbon emissions [4]. Translating the general present tense to match the language of scientific papers [5,6]. The hydrogen evolution reaction (HER), as the core process of water splitting for hydrogen production, exhibits efficiency highly dependent on catalyst performance. Although platinum (Pt)-based noble metal catalysts demonstrate exceptional activity, their scarcity and high costs severely limit large-scale applications [7]. Consequently, designing efficient, stable, and cost-effective non-noble metal HER catalysts has emerged as a key research focus in this field.

Based on the construction strategy of catalyst active sites, modern HER catalyst systems are mainly divided into supported catalysts, intrinsic active framework materials, and self-supported catalytic systems. Among them, supported catalysts are often composed of carriers and catalytic matrices [8]. An ideal catalyst support possesses both a high specific surface area and excellent electrical conductivity, with carbon aerogels meeting these critical requirements. The limitations of carbon aerogels as an HER catalyst are mainly reflected in their lack of intrinsic activity, limited structural stability, and complicated preparation process. When used as a catalytic carrier, they have significant advantages. The high specific surface area and hierarchical pore structure of carbon aerogels can optimize the active site dispersion and mass transfer efficiency [9]. The controllable adjustment of surface chemistry and electronic structure can guide the design of an electrocatalytic active site through density functional theory (DFT), which can significantly reduce the HER overpotential and enhance the interfacial electron transfer [10]. Furthermore, the excellent chemical stability and mechanical strength of carbon aerogels can maintain long-term catalytic activity at high current density. The template-free preparation strategy and the application of renewable feedstock biomass further reduce the cost, which promotes their large-scale application in energy conversion and green catalysis [11]. For instance, Jiang et al. present a novel strategy for preparing amorphous CoO_x_ self-supported carbon aerogel (CoO_x_/PSCA) via the sol-gel method and high-temperature annealing, using potato starch as a renewable carbon source [12]. The catalyst features a three-dimensional porous architecture that effectively disperses CoO_x_ and enhances electrical conductivity. The CoO_x_/PSCA exhibits outstanding HER performance with a current density of 10 mA cm^−2^ achieved at an overpotential of 100 mV (1.0 M KOH), closely approaching commercial Pt/C catalyst (88 mV), while demonstrating excellent long-term stability. The superior electrocatalytic activity originates from the synergistic effects between highly active sites of amorphous CoO_x_ clusters and the conductive carbon aerogel matrix.

In recent years, transition metal sulfides have become a research hotspot for HER catalysts owing to their unique electronic structures, tunable active sites, and low costs. However, the catalytic performance of single-component sulfides is often constrained by insufficient exposure to active sites, poor conductivity, and inert basal planes [13]. For example, MoS_2_ primarily exhibits HER activity at edge sites, while its basal planes remain catalytically inactive. To address these limitations, heterojunction construction has proven effective in enhancing catalytic performance; the electronic coupling effect at heterointerfaces optimizes hydrogen adsorption-free energy (ΔG_H*_), while synergistically improving conductivity and stability [14,15,16]. Hu et al. developed an innovative heterostructure by growing MoS_2_-CoS_2_ nanosheets on highly conductive reduced graphene oxide (MoS_2_-CoS_2_/RGO) through a facile synthesis strategy. The strong electronic interaction at the MoS_2_/CoS_2_ interface induces charge redistribution, thereby optimizing intermediate adsorption–desorption kinetics. The resulting catalyst achieves low overpotentials of 67 mV (1.0 M KOH) and 95 mV (0.5 M H_2_SO_4_) with outstanding stability [17].

Herein, this work proposes the integration of a Co_9_S_8_/FeCoS_2_ heterostructure onto bamboo fiber-derived nitrogen-doped porous carbon (BFPC) to synergistically leverage the electronic modulation advantages of the heterojunction and the structural merits of the porous carbon carrier. BFPC has properties similar to carbon aerogel. The strong electronic interaction at the Co_9_S_8_/FeCoS_2_ interface facilitates efficient electron transfer and exposes additional active sites through interfacial-engineered heterostructure formation, while the highly conductive BFPC carbon quasi-aerogel synergistically accelerates charge transport. This work not only provides new insights into the design of high-performance HER catalysts but also expands the scientific foundation for the application of biomass-derived carbon materials in energy conversion systems.

## 2. Results and Discussion

**Figure 1:**
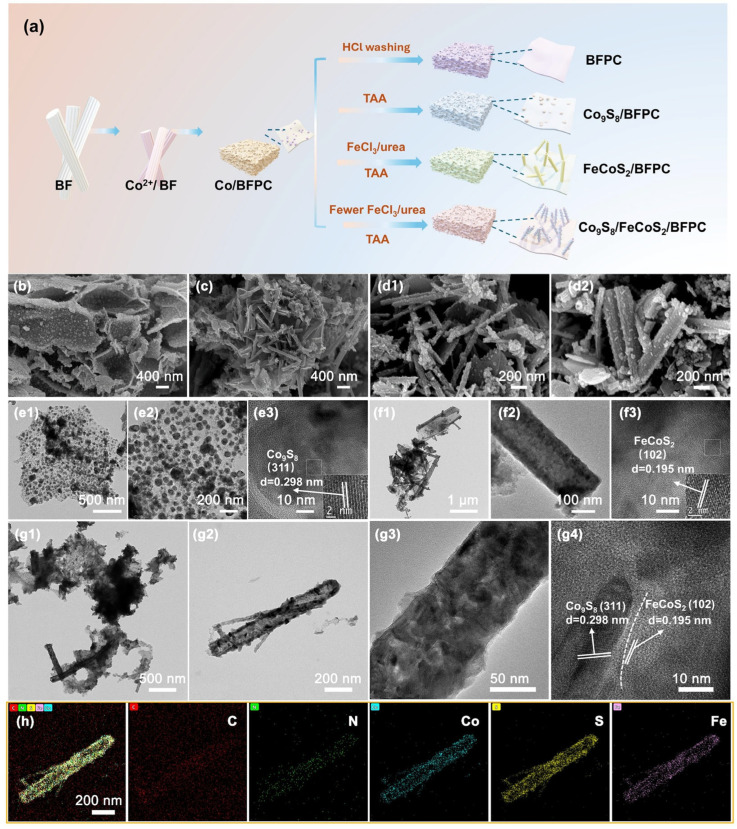
(**a**) Illustration of the synthesis process for Co_9_S_8_/FeCoS_2_/BFPC, FeCoS_2_/BFPC, Co_9_S_8_/BFPC, and BFPC. (**b**) FESEM of Co_9_S_8_/BFPC. (**c**) FESEM of FeCoS_2_/BFPC. (**d1**,**d2**) FESEM of Co_9_S_8_/FeCoS_2_/BFPC. (**e1**,**e2**) TEM and (**e3**) HRTEM of Co_9_S_8_/BFPC. (**f1**,**f2**) TEM and (**f3**) HRTEM of FeCoS_2_/BFPC. (**g1**–**g3**) TEM, (**g4**) HRTEM, and (**h**) EDS mappings of Co_9_S_8_/FeCoS_2_/BFPC.

Unlike classical aerogels synthesized via sole-gel polymerization, the 3D porous structure of BFPC arises from the natural bamboo cellulose pyrolysis and subsequent activation. In the synthesis process of Co_9_S_8_/FeCoS_2_/BFPC (Figure 1a), the precursor Co/BFPC is initially synthesized for all catalyst samples. Brunauer–Emmett–Teller (BET) analysis reveals that the BFPC material exhibits a high specific surface area of 771.58 m^2^ g^−1^ with abundant porosity distributed throughout its structure, demonstrating characteristics typical of carbon quasi-aerogel materials [18]. As previously reported, the bamboo fiber cloth (BFC) is first immersed in a Co(NO_3_)_2_ solution, allowing Co^2+^ ions to be uniformly distributed within the bamboo fiber support. The dried fiber cloth is subsequently subjected to high-temperature pyrolysis under an Ar atmosphere to obtain metallic Co-loaded porous carbon (Co/BFPC). The metallic Co is then removed through hydrochloric acid washing to produce BFPC carbon quasi-aerogel. Co_9_S_8_/BFPC is obtained by directly sulfidizing Co/BFPC. For further modification, the Co/BFPC samples are immersed in FeCl_3_ solutions with varying concentrations. During this process, the strong acidity from FeCl_3_ hydrolysis causes partial dissolution of metallic Co on the BFPC surface, resulting in the release of Co^2+^ ions into the solution. Subsequently, a urea-containing ethanol solution is introduced. Urea is employed to establish an alkaline environment in the solution, while ethanol is added to enhance the dispersion stability of components within the reaction system. The hydrothermal reaction is conducted at 90 °C, where BFPC undergoes partial hydrolysis and subsequently recombines with metal ions in the solution to form C- and N-enriched rod-shaped metal structures. The alkaline environment generated by urea simultaneously etches the recombined rod-shaped metal compounds, thereby inducing the formation of hollow-structured metal rods. The resulting product is then subjected to sulfidation treatment at 400 °C. Depending on the concentration of FeCl_3_ introduced, the dissolution efficiency of Co varies significantly, ultimately yielding two distinct products: FeCoS_2_/BFPC and Co_9_S_8_/FeCoS_2_/BFPC. In the synthesis of FeCoS_2_/BFPC, FeCl_3_ hydrolysis achieves complete dissolution of metallic Co from the Co/BFPC precursor, resulting in a smooth FeCoS_2_ surface devoid of particulate residues. Conversely, during the preparation of Co_9_S_8_/FeCoS_2_/BFPC, partial dissolution of Co occurs under FeCl_3_ hydrolysis. The residual metallic Co is etched and decomposed in the urea-induced alkaline environment, followed by redeposition onto the rod surfaces. During the sulfidation stage, the undissolved metallic Co is transformed into Co_9_S_8_ nanoparticles, which are uniformly dispersed on the FeCoS_2_ surface, thereby constructing a heterostructure.

The morphologies of BFC and BFPC are characterized by FESEM (Figure S1). Significant morphological differences are observed between BFC and BFPC before and after the crushing process. As reported in previous studies, crushed BFPC exhibits an increased specific surface area and higher pore density, forming a carbon quasi-aerogel structure that facilitates its application as a catalyst support [19]. These structural advantages are attributed to enhanced catalyst dispersion and improved accessibility of active sites [20]. The morphologies of Co_9_S_8_/BFPC, FeCoS_2_/BFPC, and Co_9_S_8_/FeCoS_2_/BFPC are further characterized by field emission scanning electron microscope (FESEM). As shown in Figure 1b, the overall morphology of Co_9_S_8_/BFPC closely resembles that of BFPC, with Co_9_S_8_ particles uniformly dispersed on the BFPC surface. This homogeneous distribution indicates effective infiltration of Co^2+^ into the BF during the impregnation process. In contrast, FeCoS_2_ and Co_9_S_8_/FeCoS_2_ are primarily observed as hollow rods on the BFPC surface (Figure 1(c–d2)). A distinct difference is noted between FeCoS_2_/BFPC and Co_9_S_8_/FeCoS_2_/BFPC. FeCoS_2_ exhibits a relatively smooth surface, while Co_9_S_8_/FeCoS_2_ displays numerous surface-adhered particles. Comparative analysis confirms that the rod-shaped framework corresponds to FeCoS_2_, with Co_9_S_8_ particles uniformly dispersed on its surface, forming a heterostructure [21].

The microstructures are further investigated by transmission electron microscopy (TEM) and energy-dispersive X-ray spectroscopy (EDS) [22]. For Co_9_S_8_/BFPC, the Co_9_S_8_ particles are uniformly distributed on the BFPC carbon quasi-aerogel surface (Figure 1(e1,e2)). High-resolution TEM (HRTEM) analysis reveals lattice fringes with a spacing of 0.298 nm, corresponding to the (311) plane of Co_9_S_8_ (Figure 1(e3)). For FeCoS_2_/BFPC, FeCoS_2_ rods are observed overlapping with BFPC, and no residual particles are detected on BFPC, confirming the complete dissolution of Co (Figure 1(f1,f2)) [23]. The hollow FeCoS_2_ rods exhibit smooth surfaces, with HRTEM-measured lattice spacings of 0.195 nm, consistent with the (102) plane of FeCoS_2_ (Figure 1(f3)) [24]. Co_9_S_8_/FeCoS_2_/BFPC’s TEM images reveal particle-free BFPC surfaces, suggesting that undissolved Co is removed from BFPC during the hydrothermal process under urea-mediated alkaline conditions and subsequently redeposited onto the rod surfaces (Figure 1(g1–g3)). Distinct particles are observed on the hollow Co_9_S_8_/FeCoS_2_ rods, confirming the presence of Co_9_S_8_. HRTEM analysis of Co_9_S_8_/FeCoS_2_/BFPC demonstrates two distinct lattice fringes with spacings of 0.298 nm and 0.195 nm, corresponding to the (311) plane of Co_9_S_8_ and the (102) plane of FeCoS_2_, respectively (Figure 1(g4)). This dual-phase lattice alignment provides direct evidence for the formation of a Co_9_S_8_/FeCoS_2_ heterostructure [25].

The elemental compositions of the Co_9_S_8_/BFPC, FeCoS_2_/BFPC, and Co_9_S_8_/FeCoS_2_/BFPC composites are confirmed by EDS analysis to align with their expected structural configurations. In the EDS spectrum of Co_9_S_8_/BFPC (Figure S2a), distinct signals of Co and S are detected, while stable C and N signals from the BFPC substrate are observed. At the same time, XPS analysis of the Co9S8/BFPC sample revealed atomic percentages of Co (6.34%) and S (5.46%), close to the theoretical 9:8 for Co_9_S_8_. These results confirm the successful integration of Co_9_S_8_ with the carbon-based support [26]. In Figure S2b, the EDS profile of FeCoS_2_/BFPC reveals the coexistence of C, N, Fe, Co, and S elements, with a Fe/Co atomic ratio of approximately 1:1, which matches the stoichiometry of FeCoS_2_ (Table S1). In the composite Co_9_S_8_/FeCoS_2_/BFPC, all constituent elements (C, N, Co, Fe, S) are prominently identified, and uniform elemental distribution is observed without detectable segregation, indicating homogeneous loading and interfacial contact between Co_9_S_8_ and FeCoS_2_ on the BFPC support (Figure 1h). The structural integrity of the composite is further corroborated by the consistent C and N signals from the BFPC matrix. Furthermore, the presence of C and N within the metallic rods is demonstrated to enhance the catalytic stability of the active phases during reactions by suppressing metal dissolution [27,28].

**Figure 2:**
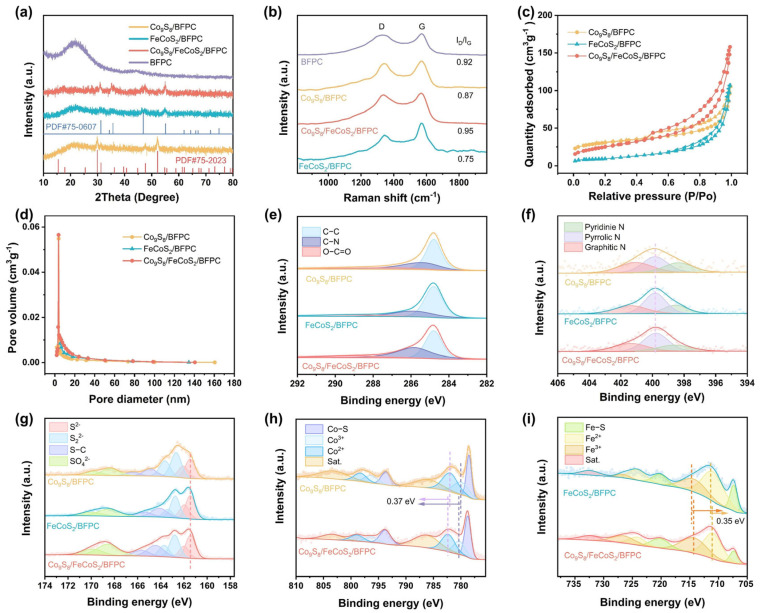
(**a**) XRD, (**b**) Raman spectra of BFPC, Co_9_S_8_/BFPC, FeCoS_2_/BFPC, and Co_9_S_8_/FeCoS_2_/BFPC. (**c**) N_2_ adsorption–desorption isotherm, (**d**) pore size distribution curves, (**e**) C1s XPS spectra, (**f**) N1s XPS spectra, (**g**) S 2p XPS spectra of Co_9_S_8_/BFPC, FeCoS_2_/BFPC, and Co_9_S_8_/FeCoS_2_/BFPC. (**h**) Co 2p XPS spectra of Co_9_S_8_/BFPC and Co_9_S_8_/FeCoS_2_/BFPC. (**i**) Fe 2p XPS spectra of FeCoS_2_/BFPC and Co_9_S_8_/FeCoS_2_/BFPC.

The crystalline structures of the samples are investigated by X-ray diffraction (XRD) [29]. As shown in Figure S3, the XRD pattern of Co/BFPC exhibits three prominent diffraction peaks at 2θ = 44.216°, 51.522°, and 75.853°, which are assigned to the (111), (200), and (220) crystallographic planes of metallic Co (PDF#15-0806). In contrast, the XRD pattern of BFPC shows no detectable metallic diffraction peaks, with only characteristic peaks of carbon materials observed, confirming that metallic Co is completely removed, leaving solely the porous carbon framework (Figure 2a). For Co_9_S_8_/BFPC, synthesized by direct sulfidation of Co/BFPC, distinct diffraction peaks are identified at 2θ = 29.888°, 31.25°, 47.658°, and 52.168°, corresponding to the (311), (222), (511), and (440) planes of Co_9_S_8_ (PDF#75-2023). In the case of FeCoS_2_/BFPC, characteristic peaks are observed at 2θ = 30.7°, 35.17°, 46.351°, and 54.581°, which are indexed to the (100), (101), (102), and (110) planes of FeCoS_2_ (PDF#75-0607). The XRD pattern of Co_9_S_8_/FeCoS_2_/BFPC reveals coexisting diffraction peaks from both Co_9_S_8_ and FeCoS_2_ phases. However, the diffraction intensities of FeCoS_2_ are slightly stronger than those of Co_9_S_8_, which is attributed to the lower relative content of Co_9_S_8_ compared to FeCoS_2_ in the composite [30].

The structural characteristics of BFPC, Co_9_S_8_/BFPC, FeCoS_2_/BFPC, and Co_9_S_8_/FeCoS_2_/BFPC composites are analyzed by Raman spectroscopy, as shown in Figure 2b [31]. All samples exhibit two broad peaks at 1350 and 1580 cm^−1^, corresponding to the characteristic D-band and G-band of carbon materials, respectively. The D-band is attributed to lattice defects and disordered carbon structures, whereas the G-band arises from in-plane vibrational modes of graphitic crystallites. The graphitization degree of carbon is quantified by the intensity ratio (I_D_/I_G_). Notably, the Co_9_S_8_/FeCoS_2_/BFPC composite demonstrates the highest I_D_/I_G_ ratio (0.95) among the samples, introducing additional structural defects and disordered domains [32]. This enhanced defect density in Co_9_S_8_/FeCoS_2_/BFPC is proposed to create abundant active sites, which synergistically improve the electrocatalytic HER activity by facilitating reactant adsorption and charge transfer processes [33].

The porosity characteristics of the samples are evaluated through nitrogen adsorption–desorption isotherm measurements. As shown in Figure 2c,d, the isotherms of Co_9_S_8_/BFPC, FeCoS_2_/BFPC, and Co_9_S_8_/FeCoS_2_/BFPC are classified as Type IV with distinct hysteresis loops, indicating mesoporous-dominated structures [34]. Their specific surface areas are calculated to be 86.7, 33.8, and 262.7 m^2^ g^−1^, respectively (Table S2). The pore size distribution profiles reveal that the majority of pores are concentrated in the 20–60 nm range, suggesting a hierarchical porosity dominated by mesopores (2–50 nm) and macropores (>50 nm). Mesopores provide abundant exposed active sites while facilitating electrolyte penetration, ensuring full wetting of the catalytic surface and optimal contact between active sites and reactants [35]. At the same time, macropores serve as mass transport highways, accelerating the diffusion of generated H_2_ bubbles and ionic species during reactions [36,37]. This reduces mass transfer resistance, mitigates bubble accumulation, and improves structural stability by alleviating volumetric strain during catalytic cycles, thereby preventing mechanical degradation of the material [38].

Figure 2e presents the C 1s XPS spectra, comparing the carbon chemical states of Co_9_S_8_/BFPC, FeCoS_2_/BFPC, and Co_9_S_8_/FeCoS_2_/BFPC. All catalysts exhibit distinct peaks attributed to C-C (284.8 eV), C-N (285.6 eV), and O-C=O (288.5 eV), confirming the presence of nitrogen doping and oxygen-containing functional groups in the carbon framework [39]. The consistent appearance of C-N peaks further verifies the successful integration of nitrogen atoms into the carbon matrix, which likely contributes to the formation of catalytically active sites [33,40]. Figure 2f displays the N 1s XPS spectra of the three catalysts, illustrating the distribution of nitrogen-doped configurations: pyridinic-N (398.3 eV), pyrrolic-N (400.1 eV), and graphitic-N (401.3 eV) [20]. As illustrated in Figure 2g, the S 2p XPS spectra of Co_9_S_8_/BFPC, FeCoS_2_/BFPC, and Co_9_S_8_/FeCoS_2_/BFPC composites exhibit distinct spin–orbit split doublets (2p_3/2_ and 2p_1/2_) [41]. Deconvolution analysis reveals four sulfur species: S^2−^ (161.4 and 162.1 eV), S_2_^2−^ (162.8 and 163.8 eV), S-C (164.5 and 165.8 eV), and SO_4_^2−^ [42]. The XPS analysis of Co 2p and Fe 2p reveals that the superior HER performance of the Co_9_S_8_/FeCoS_2_/BFPC composite catalyst originates from synergistic chemical state modulation and interfacial electronic interactions. As shown in Figure 2h and Figure S4, the Co 2p XPS spectra of Co_9_S_8_/BFPC and FeCoS_2_/BFPC exhibit four distinct chemical states: metallic Co-S bonding (778.5 eV and 793.8 eV), Co^2+^ (780.4 eV and 796.2 eV), Co^3+^ (782.3 eV and 798.9 eV), and satellite peaks [43,44]. The XPS analysis of the Co 2p spectra in the Co_9_S_8_/FeCoS_2_/BFPC composite reveals a uniform positive shift of 0.37 eV in the binding energy positions for both Co^3+^ and Co^2+^ species compared to Co_9_S_8_/BFPC. This phenomenon suggests electron transfer from Co sites to adjacent electronegative components, resulting in optimized electronic interactions at the heterointerfaces. Fe 2p XPS spectra of FeCoS_2_/BFPC and Co_9_S_8_/FeCoS_2_/BFPC show the coexistence of multiple chemical states of Fe (Figure 2i). Fe 2p XPS spectra of FeCoS_2_/BFPC exhibit four distinct chemical states: Fe-S (707.4 and 720.3 eV), Fe^2+^ (711.3 and 724.5 eV), Fe^3+^ (714.7 and 726.8 eV), and satellite peaks [45]. In the Co_9_S_8_/FeCoS_2_/BFPC composite, the Fe^3+^ and Fe^2+^ species exhibit a negative binding energy shift of 0.35 eV compared to pristine FeCoS_2_/BFPC, attributed to electron donation from the Co_9_S_8_ phase, which increases the electron density around Fe centers [25]. The mixed oxidation states (Co^3+^/Co^2+^, Fe^3+^/Fe^2+^) create diversified active sites to synergistically optimize water dissociation and hydrogen adsorption energetics [46]. Concurrently, heterointerface-induced electron redistribution enhances charge transfer efficiency, while surface defects act as catalytic hotspots to accelerate reaction kinetics [47].

**Figure 3:**
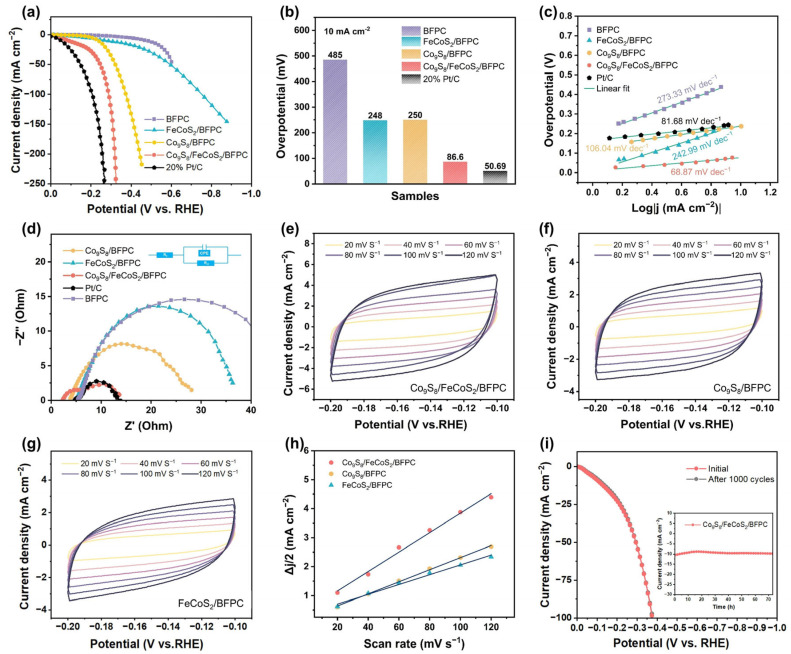
(**a**) LSV, (**b**) Overpotentials curves, (**c**) Tafel plots, and (**d**) EIS plots of 20% Pt/C, BFPC, Co_9_S_8_/BFPC, FeCoS_2_/BFPC, and Co_9_S_8_/FeCoS_2_/BFPC. Cyclic voltammograms of (**e**) Co_9_S_8_/FeCoS_2_/BFPC, (**f**) Co_9_S_8_/BFPC, and (**g**) FeCoS_2_/BFPC. (**h**) C_dl_ of Co_9_S_8_/BFPC, FeCoS_2_/BFPC, and Co_9_S_8_/FeCoS_2_/BFPC. (**i**) Chronopotentiometry test and LSV curves before and after 1000 cyclic voltammetry of Co_9_S_8_/FeCoS_2_/BFPC.

The HER performances of BFPC, Co_9_S_8_/BFPC, FeCoS_2_/BFPC, and Co_9_S_8_/FeCoS_2_/BFPC catalysts are evaluated in alkaline media (1 mol L^−1^ KOH) using a three-electrode system. As shown in Figure 3a,b, the linear sweep voltammetry (LSV) curves, and the Co_9_S_8_/FeCoS_2_/BFPC heterostructure exhibits an overpotential of 86.6 mV at −10 mA cm^−2^, whereas BFPC requires a significantly higher overpotential of 480 mV at the same current density. This stark contrast confirms that the primary catalytic activity originates from the loaded metal sulfide phases rather than the carbon support. To emphasize the critical role of heterostructure engineering, comparative electrochemical tests are conducted for Co_9_S_8_/BFPC and FeCoS_2_/BFPC. The LSV comparison reveals that Co_9_S_8_/FeCoS_2_/BFPC retains a distinct performance advantage over its single-phase counterparts. Although a gap persists compared to commercial Pt/C, Co_9_S_8_/FeCoS_2_/BFPC demonstrates competitive HER activity. The Tafel slope, which reflects the rate-determining step and intrinsic catalytic efficiency, is analyzed to probe reaction kinetics. A lower Tafel slope indicates optimized adsorption–desorption capabilities for H*, enabling faster kinetics [48]. As shown in Figure 3c, Co_9_S_8_/FeCoS_2_/BFPC achieves a Tafel slope of 68.67 mV dec^−1^, significantly lower than those of Co_9_S_8_/BFPC (106.04 mV dec^−1^) and FeCoS_2_/BFPC (242.99 mV dec^−1^). This enhancement is attributed to the heterointerface-driven modulation of H* adsorption–desorption free energy, which reduces the reaction energy barrier and accelerates charge transfer kinetics [49]. Nyquist plots further reveal the charge transfer resistance (R_ct_) differences among the catalysts [50]. In addition to Pt/C (R_ct_ = 8.9 Ω), the Co_9_S_8_/FeCoS_2_/BFPC heterostructure exhibits the smallest semicircle diameter (R_ct_ = 12.7 Ω), indicating superior interfacial charge transfer efficiency (Figure 3d). This is ascribed to the synergistic electronic interaction between Co_9_S_8_ and FeCoS_2_, where heterointerface reconstruction optimizes H* adsorption energetics, while the highly conductive BFPC substrate facilitates rapid electron transport [51]. In contrast, FeCoS_2_/BFPC and Co_9_S_8_/BFPC show higher R_ct_ values of 22.4 Ω and 38.7 Ω, respectively, due to limited active sites and weaker interfacial interactions in single-phase sulfides [52]. The unmodified BFPC substrate, lacking catalytic sites and efficient electron pathways, demonstrates the highest charge transfer resistance. The double-layer capacitance (C_dl_) and electrochemical active surface area (ECSA) of Co_9_S_8_/BFPC, FeCoS_2_/BFPC, and Co_9_S_8_/FeCoS_2_/BFPC heterostructures are systematically analyzed in Figure 3e–h. The cyclic voltammetry (CV) curves of Co_9_S_8_/FeCoS_2_/BFPC are characterized by the highest current response within the identical potential window, with current density increasing linearly with scan rate, indicative of superior capacitive behavior. The C_dl_ value of Co_9_S_8_/FeCoS_2_/BFPC is calculated as 3.36 mF cm^−2^, significantly higher than those of Co_9_S_8_/BFPC (2.09 mF cm^−2^) and FeCoS_2_/BFPC (1.69 mF cm^−2^), confirming a substantially enlarged ECSA. This enhancement is attributed to the heterointerface between Co_9_S_8_ and FeCoS_2,_ which optimizes charge distribution through interfacial electronic coupling, thereby increasing the density of catalytically accessible sites [53]. The hierarchical porous architecture of the BFPC as support promotes uniform dispersion of active phases, further exposing abundant edge sites [54]. The high active site density of Co_9_S_8_/FeCoS_2_/BFPC is identified as a critical factor contributing to its superior HER kinetics. The cycling stability of catalysts is a critical metric for evaluating their practical applicability [55]. As presented in Figure 3i, the long-term durability of the Co_9_S_8_/FeCoS_2_/BFPC heterostructure catalyst is systematically assessed through 1000 cyclic voltammetry cycles and chronoamperometry testing under constant potential. At a fixed overpotential (−10 mA cm^−2^), the current density of Co_9_S_8_/FeCoS_2_/BFPC remains stable for 73 h of continuous operation, with no significant current fluctuations or abrupt decay observed, demonstrating exceptional structural integrity without active site detachment or framework collapse. Furthermore, the post-stability LSV curve overlaps closely with the initial profile, confirming negligible degradation in catalytic activity after prolonged cycling.

**Figure 4:**
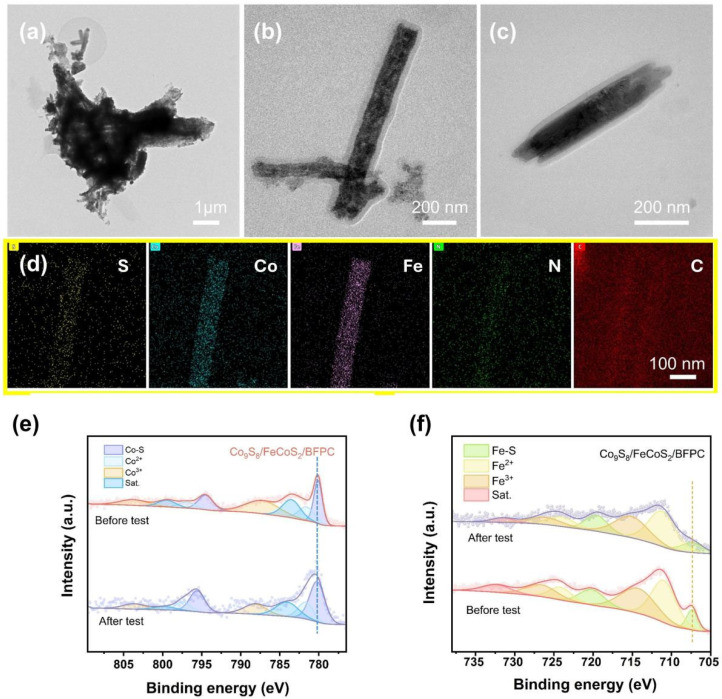
(**a**–**c**) TEM, (**d**) EDS mappings, (**e**) Co 2p XPS spectra, and (**f**) Fe 2p XPS spectra of Co_9_S_8_/FeCoS_2_/BFPC after chronopotentiometry test.

Post-stability characterization of the cycled Co_9_S_8_/FeCoS_2_/BFPC catalyst is systematically conducted through TEM, XRD, and XPS analyses. As shown in Figure 4a–d the morphology and elemental composition remain virtually unchanged after cycling. Notably, distinct lattice fringes are not observed on the post-cycled catalyst surface, which can be attributed to the in situ formation of an amorphous passivation layer during the catalytic process [56]. This self-limiting surface reconstruction serves as a protective barrier to prevent further structural degradation of the catalytic core while simultaneously enhancing operational stability through kinetic modulation [57]. The XPS spectra exhibit negligible shifts in binding energies, demonstrating exceptional structural integrity throughout the catalytic process (Figure 4e,f) [58]. Notably, the attenuated XRD peak intensities are ascribed to the formation of a stable passivation layer through electrolyte ion interaction with the catalyst surface (Figure S5) [59]. This passivation layer effectively shields the underlying active phases from deep structural degradation while maintaining catalytic functionality through selective permeability to reactants [60].

## 3. Conclusions

In this study, a Co_9_S_8_/FeCoS_2_ heterostructure catalyst supported on bamboo fiber-derived nitrogen-doped porous carbon is successfully constructed via a synergistic hydrothermal carbonization strategy, demonstrating exceptional alkaline HER performance. The carbon quasi-aerogel BFPC support, with its high surface area, three-dimensional conductive network, and nitrogen-doping characteristics, effectively optimizes electronic transport pathways and active site dispersion while facilitating mass transfer. The strong electronic interaction at the Co_9_S_8_/FeCoS_2_ interface facilitates efficient electron transfer and exposes additional active sites through interfacial-engineered heterostructure formation. Experimental results reveal that the catalyst achieves a current density of 10 mA cm^−2^ at an overpotential of 86.6 mV in alkaline media, accompanied by a low Tafel slope of 68.87 mV dec^−1^, along with outstanding long-term stability over 73 h. This work not only elucidates the synergistic mechanisms between carbon substrates and heterostructures in catalytic optimization but also provides innovative insights for designing cost-effective, high-performance, non-precious-metal-based HER catalysts.

## 4. Materials and Methods

### 4.1. Materials

Bamboo fiber cloth (BF) and carbon cloth (CC) were purchased from HuaHeng company. Nafion dispersion (5 wt%) were purchased from Dupont company. All chemicals, including cobalt nitrate hexahydrate (Co(NO_3_)_2_·6H_2_O), ethanol (99.9%), potassium hydroxide (KOH), iron(III) chloride (FeCl_3_·9H_2_O), urea (CH_4_N_2_O), nickel foam, thioacetamide (TAA, C_2_H_5_NS) were purchased from Rhawn company. The reagents used above were analytically pure, and the water was distilled.

### 4.2. Synthesis of Co/BFPC, BFC and BFPC

BF used in kitchens was first treated with deionized water and ethanol, followed by air-drying. After drying, the BF was cut into square pieces of appropriate dimensions (2 cm × 3 cm) and immersed in 20 mL 0.5 mol L^−1^ Co(NO_3_)_2_·6H_2_O solution for further processing. Following immersion, the samples were dried at 60 °C for 10 h. Both the treated BF and untreated BF were then transferred to a tubular furnace, where they were annealed at 800 °C under an Ar atmosphere for 3 h. After cooling, the resulting materials were labeled as Co/BFPC and BFC, respectively. To remove metallic cobalt, the Co/BFPC sample was subsequently soaked in 2 M HCl solution for 12 h. Finally, the sample was thoroughly rinsed with deionized water and ethanol, followed by drying at 80 °C for 10 h to obtain purified BFPC.

### 4.3. Synthesis of Co_9_S_8_/BFPC, FeCoS_2_/BFPC and Co_9_S_8_/FeCoS_2_/BFPC

Initially, 50 mg of Co/BPC and 0.2 mmol FeCl_3_·6H_2_O were dissolved in 14 mL deionized water followed by 30-min ultrasonication. Subsequently, 3 mmol urea and 26 mL ethanol were added to the mixture under vigorous stirring until complete dissolution. The solution was then transferred into a Teflon-lined autoclave and maintained at 90 °C in a blast drying oven for 5 h. The resulting product was thoroughly washed with deionized water and ethanol and then dried at 60 °C in the blast drying oven. For sulfurization, the obtained sample and thioacetamide (mass ratio 1:10) were separately placed in a tubular furnace and annealed at 400 °C for 3 h under Ar atmosphere to obtain Co_9_S_8_/FeCoS_2_/BFPC. For comparative studies, two control samples were prepared. FeCoS_2_/BFPC was synthesized using 0.5 mmol FeCl_3_·6H_2_O while keeping other parameters identical, and Co_9_S_8_/BFPC was obtained by direct sulfurization of Co/BFPC under the same sulfidation conditions.

### 4.4. Electrochemical Measurements

The electrochemical characterizations of catalyst materials were conducted using a CHI 660e electrochemical workstation (Chenhua, Shanghai, China) at room temperature within a three-electrode system. The catalysts were loaded onto nickel foam as the working electrode for electrochemical testing. A mercury oxide electrode was used as the reference electrode, 1.0 M KOH served as the acidic electrolyte, and blank carbon cloth was used as the counter electrode. Before measuring the HER activity, cyclic voltammetry (CV) was performed in the potential range from −0.9 to 1.2 V (vs. SCE) at a scan rate of 100 mV s^−1^ for 200 cycles to stabilize the catalyst surface and obtain a stable CV curve. The HER activity of the catalyst was measured by linear sweep voltammetry (LSV) at a scanning speed of 2 mV s^−1^. Tafel plots were derived from LSV at low overpotentials and fitted to the Tafel equation: η = b log j + a, where η is the overpotential, j is the current density, and b is the Tafel slope. Electrochemical impedance spectroscopy (EIS) was conducted at 80 mV (vs. RHE) using a 10 mV amplitude AC signal in the frequency range from 100 kHz to 0.1 Hz. The solution resistance obtained from the Nyquist plot was used to compensate for the ohmic loss, and all results were iR-corrected. Electrochemical double-layer capacitance (C_dl_) was measured by CV performed in the non-Faradaic region (0.161 to 0.261 V (vs. SCE)) at different scan rates (20, 40, 60, 80, 100, and 120 mV s^−1^) to determine the electrochemical active surface area (ECSA) in the electrolyte. The stability tests were evaluated by potential cycling from −1.126 to −0.126 V (vs. RHE) at 100 mV s^−1^ and amperometric i-t for 73 h.

## Data Availability

The original contributions presented in this study are included in the article/Supplementary Materials. Further inquiries can be directed to the corresponding authors.

## Supplementary Materials

The following supporting information can be downloaded at: https://www.mdpi.com/article/10.3390/gels11060390/s1, Figure S1. FESEM of (a,b) BFC and (c,d) BFPC. Figure S2. EDS mappings of (a) Co9S8/BFPC and (b) FeCoS2/BFPC. Figure S3. XRD of Co/BFPC. Figure S4. Fe 2p XPS spectra of FeCoS2/BFPC. Figure S5. XRD of Co9S8/FeCoS2/BFPC after stability test. Table S1. Fe and Co content of as-prepared samples. Table S2. N2 adsorption-desorption of as-prepared samples.
