# Supplementary material for: Bi-Interfacial Electron Modulation in Co9S8/FeCoS2 Heterostructures Anchored on Bamboo-Derived Carbon Quasi-Aerogel for High-Performance Hydrogen Evolution
Source: Gels. 2025 May 25;11(6):390. doi: 10.3390/gels11060390 (PMC12192258; doi:10.3390/gels11060390)
Supplement: Supplementary file 1 [file gels-11-00390-s001.zip › gels-3595827-supplementary.pdf]

Bi-interfacial electron modulation in $Co_9S_8/FeCoS_2$ heterostructures anchored on bamboo-derived carbon quasi-aerogel for high-performance hydrogen evolution

Wenjing He[1, 2], Jinliang Cao[1, *], Xinliang Zhou[2], Ning Zhang[2], Yuzhu Qi[2], Jin Li[2], Naiteng Wu[2, *] and Xianming Liu[2,]

[1] School of Chemistry and Chemical Engineering, Henan Polytechnic University, Jiaozuo, 454000, China; hewenjing628@126.com (W.H.);

[2] College of Chemistry and Chemical Engineering, Henan Key Laboratory of Function-Oriented Porous Materials, Luoyang Normal University, Luoyang 471934, P. R. China; zxlly0904@163.com (X.Z.); zn3692580147@163.com (N.Z.); Qiyz5755@163.com (Y.Q.); lijin1986@lynu.edu.cn (J. L.); myclxm@163.com (X.L.)

Correspondence: caojianliang@hpu.edu.cn (J. C.); wunaiteng@lynu.edu.cn (N. W.);

Materials characterizations

The morphology and structures of the as-prepared samples were characterized by a scanning electron microscope (SEM, Sigma 500, Zeiss Company) and a transmission electron microscope (TEM, Talos F200S, 200 kV, FEI Company). The X-ray diffraction (XRD) analysis was performed on a Bruker D8 (Bruker Company,) with Cu Kα radiation to determine the crystal structure of the samples. X-ray photoelectron spectroscopy with Al Kα radiation (XPS, K-Alpha, Thermo Fisher Scientific) was carried out to determine the valence state of the samples. The XPS calibration procedure was based on C1s 284.8 eV. The presence of carbon was investigated by using a Raman spectrometer with an excitation laser beam wavelength of 633 nm (LabRAM Aramis, HORIBA Jobin-Yvon Company). Elemental analyses of catalyst samples were carried out using an inductively coupled plasma optical emission spectrometer (ICP-OES, Scientific iCAP 7600 Duo, Thermo Fisher Scientific, Germany). Specific surface area and pore size distribution measurements

were performed using a Quadrasorb SI-3MP instrument by N_2 adsorption at 77 K.

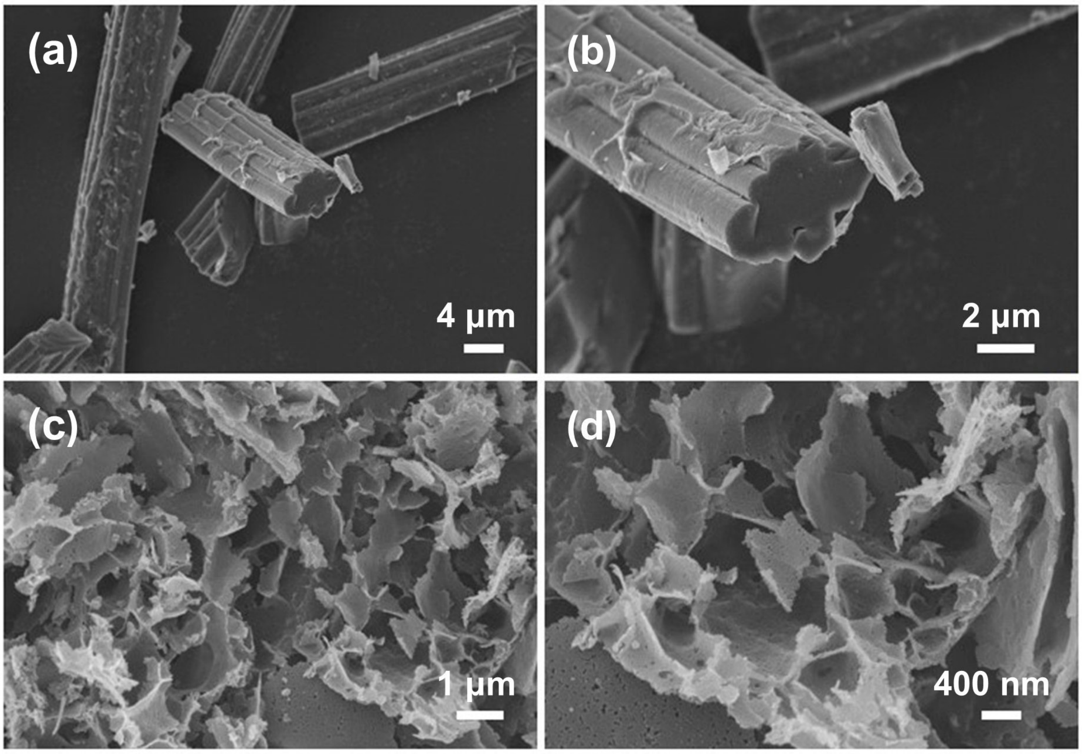

Fig. S1 FESEM of (a, b) BFC and (c, d) BFPC

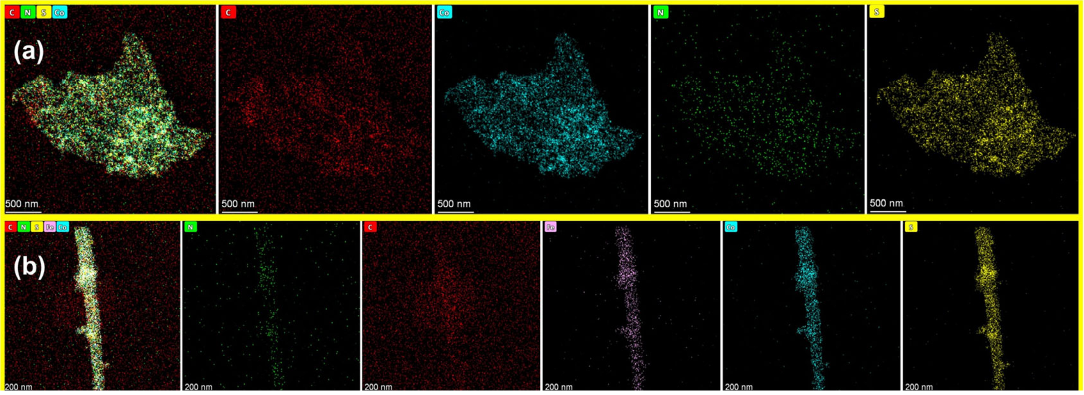

Fig. S2 EDS mappings of (a) Co_9S_8/BFPC and (b) $FeCoS_2$/BFPC

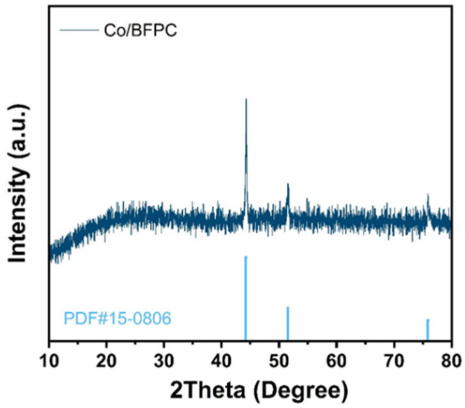

Fig. S3 XRD of Co/BFPC

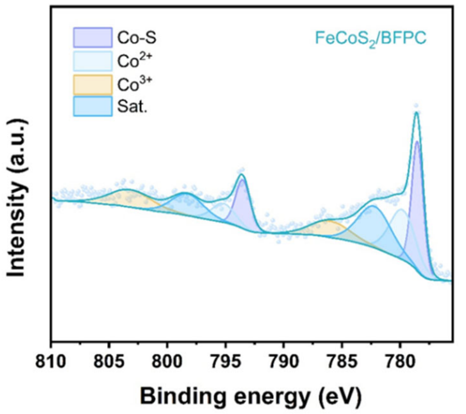

Fig. S4 Fe 2p XPS spectra of $FeCoS_2$/BFPC

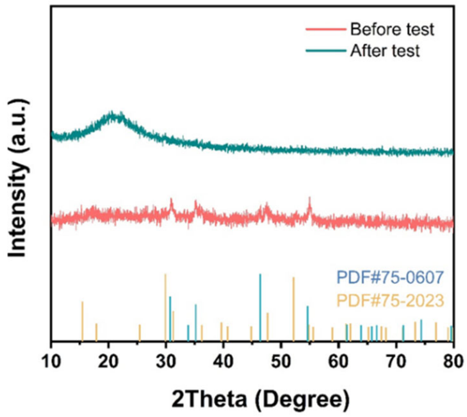

Fig. S5 XRD of Co_9S_8/$FeCoS_2$/BFPC after stability test

Table S1 Fe and Co content of as-prepared samples

	Co_9S_8/BFPC	$FeCoS_2$/BFPC	Co_9S_8/$FeCoS_2$/BFPC
Fe（Wt %）	0	2.33	1.49
Co（Wt %）	9.2	2.33	3.36

Table S2 N_2 adsorption-desorption of as-prepared samples

	Co_9S_8/BFPC	$FeCoS_2$/BFPC	Co_9S_8/$FeCoS_2$/BFPC
BET surface area (m^2 g^{-1})	86.74	33.86	262.72
Micropore volume (cm^3 g^{-1})	0.24	0.16	0.54
Average pore size (nm)	11.35	19.51	8.90
